# Coeval primary and diagenetic carbonates in lacustrine sediments challenge palaeoclimate interpretations

**DOI:** 10.1038/s41598-021-86872-1

**Published:** 2021-04-12

**Authors:** Jeremy McCormack, Ola Kwiecien

**Affiliations:** 1 Department of Human Evolution, Max Planck Institute for Evolutionary Anthropology, Deutscher Platz 6, 04103 Leipzig, Germany; 2 Department of Geography and Environmental Science, Northumbria University, Newcastle upon Tyne, NE1 8ST UK

**Keywords:** Biogeochemistry, Limnology, Environmental sciences, Climate sciences, Palaeoclimate

## Abstract

Lakes are sensitive to climate change and their sediments play a pivotal role as environmental recorders. The oxygen and carbon isotope composition (δ^18^O and δ^13^C) of carbonates from alkaline lakes is featured in numerous studies attempting a quantitative reconstruction of rainfall, temperature and precipitation-evaporation changes. An often-overlooked challenge consists in the mineralogically mixed nature of carbonates themselves. We document a large variability of carbonate components and their respective distinct δ^18^O and δ^13^C values from sediments of Lake Van (Turkey) covering the last 150 kyr. The carbonate inventory consists of primary (1) inorganic calcite and aragonite precipitating in the surface-water, (2) biogenic calcite ostracod valves; and post-depositional phases: (3) dolomite forming in the sediment, and previously overlooked, (4) aragonite encrustations formed rapidly around decaying organic matter. We find a systematic relation between the lithology and the dominant deep-water carbonate phase formed recurrently under specific hydrological conditions. The presence of the different carbonates is never mutually exclusive, and the isotopic composition of each phase forms a distinctive cluster characteristic for the depth and timing of their formation. Our findings stretch the envelope of mechanisms forming lacustrine carbonates and highlight the urge to identify and separate carbonate components prior to geochemical analyses.

## Introduction

Semiarid regions such as the Mediterranean or the Tibetan Plateau are disproportionally responsive to the impact of anthropogenic climate change^1–3^. Credible projections of water availability in global warming scenarios rely on an accurate recording and understanding of past climate change. An ultimate goal of palaeoclimate investigations lies in quantifying past environmental parameters. Mass balance isotope models combining lake monitoring and sedimentary carbonate analyses facilitate this enterprise and allow for, under several assumptions, the quantitative reconstruction of atmospheric temperature^4,5^ or rainfall^6^.

Yet climate reconstructions are only as reliable as the validity of the assumptions and the quality of the proxies applied. While the oxygen and carbon isotope composition (δ^18^O and δ^13^C) of carbonates from alkaline lakes (modern and ancient) is a common climate proxy in semiarid regions^7,8^, its frequent poor or dis-agreement with carbonate-independent proxies (e.g.; pollen records) questions its reliability^9–11^. In contrast to freshwater lakes in humid areas, saline and/or alkaline lakes, typical for semiarid regions commonly have a much more variable carbonate mineral composition^12,13^. Such variations in the mineral composition can add yet another complexity to alkaline lake carbonate δ^18^O and δ^13^C records, especially when analysing the bulk carbonate composition^14^. In modern and sub-modern lake sediments diagenetic changes affecting carbonates are often unsuspected and the few studies which documented them suggest that primary water-column and diagenetic carbonates appear to be mostly mutually exclusive^15,16^. Alternatively, diagenetic processes, i.e., all physical and chemical processes following sediment deposition, may lead to a selective dissolution of carbonate minerals, thereby altering the original depositional bulk carbonate composition^17^. The issue of variable mineralogies reaches its interpretative zenith in ancient carbonates where a diagenetic overprint can modify, partly or completely, the original signal confusing environmental interpretations. Depending on their volumetric contribution, the isotopic signature of individual carbonate phases will influence the bulk record to a smaller or larger extent. As the relative contribution of carbonate phases and, subsequently their influence on bulk isotope values, may vary through time, palaeoclimatic interpretations based on bulk carbonates are prone to errors.

Environmental signals of primary carbonates and their diagenetic overprint have been studied on individual components in ancient lakes^18^. In this work primary carbonates relate to direct precipitates which can be either inorganic (e.g., surface-water precipitates) or biogenic (e.g., CaCO_3_ shells) in origin. Diagenetic carbonates relate to sedimentary post-depositional carbonate precipitation either secondary (i.e., remineralisation of deposited carbonate) or a direct precipitation of carbonate after sediment deposition (i.e., primary sensu stricto). Instrumental advances of the last decades allow for a high spatial resolution of geochemical, mineralogical and petrographical analyses, which facilitate resolving the sequence of events from original precipitation and deposition to diagenetic alteration. These analyses, while unquestionably valuable, grant snapshots of environmental conditions rather than timeseries and provide only limited insights into the high-resolution chronology of post-depositional processes.

Here we take advantage of the multi-carbonate record of alkaline Lake Van, Turkey (ICDP PALEOVAN project) and test the sensitivity of individual carbonate phases as recorders of palaeohydrological information. We document, in sediment cores collected at the Ahlat Ridge site (AR) covering the last 150 ka, an unprecedented spectrum of concurrent carbonate phases and add yet another hitherto overlooked component, exhibiting a distinct isotopic signature and occurring exclusively on biological remains under well-ventilated bottom-water conditions. We highlight the isotopic differences of concurrent deep-water carbonate phases beyond mineral-specific fractionation factors, as a result of processes involved in their precipitation and their (micro-) environment. By comparing and contrasting their individual isotopic compositions within a stratigraphic horizon and their variation within the sedimentary profile, we investigate their strength and limitations in recording palaeohydrological conditions. Further, we suggest that the sub-recent sediments of Lake Van provide a good validation for early diagenesis models. Finally, we discuss the implications of our findings for palaeoclimate interpretations and the formation of diagenetic carbonates in Lake Van and other alkaline lakes.

## Results

**Figure 1:**
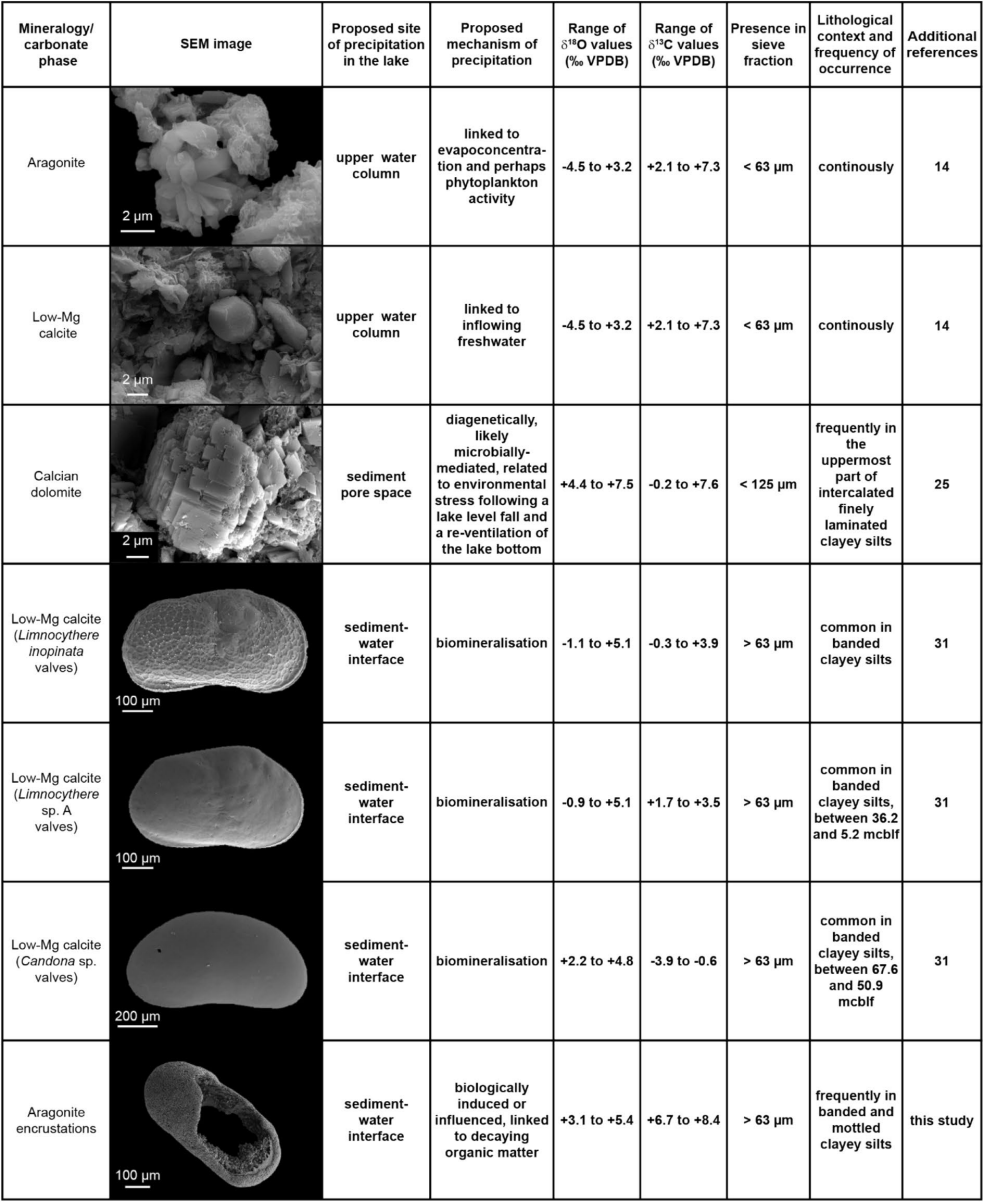
Variability of carbonate components in Lake Van with respective δ^18^O and δ^13^C isotope range. Note the high variability in δ^18^O and δ^13^C values between each carbonate phase. The additional references provide further information on the isotopic composition and environmental context of the individual carbonate components. SEM images of aragonite, low-Mg calcite and dolomite are from McCormack et al.^14^, images of ostracod valves are from McCormack et al.^31^.

**Figure 2:**
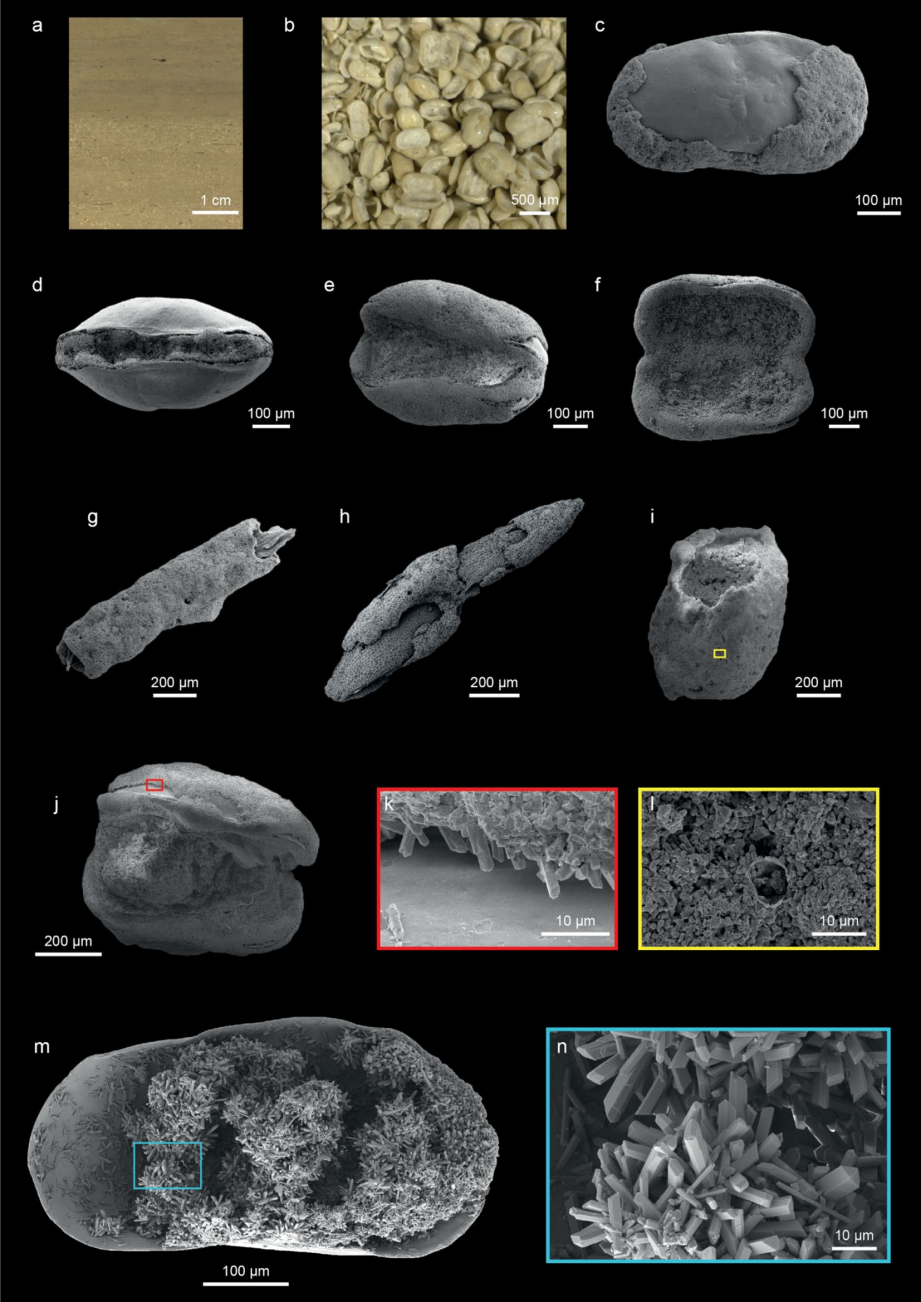
Compilation of images of diagenetic aragonite encrustations. (**a**) Aragonite encrustations occurring in high density within the Ahlat Ridge composite profile sediment (5034_2_E_11H_1, 49.3 to 53.7 cm depth), visible to the naked eye as speckled dots in high resolution core images (ICDP sampling party). Note the abrupt transition between an abundance of aragonite encrustations in the lower part of the section and their absence in the upper part. (**b**) Digital stereomicroscope photograph depicting a part of the sieved > 250 µm fraction from (5034_2_E_11H_1, 54 to 56 cm depth). The image taken using the image software ZEN 2.3 lite (https://www.zeiss.de/mikroskopie/produkte/mikroskopsoftware/zen-lite/zen-lite-download.html). Note the abundance of encrusted ostracod valves of which many are still articulated. (**c**) External lateral view of a partly encrusted ostracod valve missing the typical reticulation on the valve visible on well preserved Limnocytherinae valves typical for this depth in Lake Van (52.087 mcblf). (**d**–**f**) Articulated encrusted ostracod valves showing various stages of carapace opening. g) Tubular encrustation around a still encapsulated unidentified biological remain. (**h**) Partly encrusted biological remain. (**i**) hollow encrusted cast structure with missing original substrate on which aragonite precipitated. The yellow area is magnified in (**l**). (**j**) Articulated ostracod valves with a smaller carapace stacked inside. The red area is magnified in (**k**). (**k**) Magnification showing larger aragonite crystals growing in the hollow area between valve and encrustation. (**l**) Magnification showing small aragonite crystals intercalated with clay minerals in the surface of an encrusted tube (**i**). (**m**) Large euhedral aragonite crystals precipitated within a previously enclosed carapace. Blue area is magnified in (**n**). (**n**) Magnification of the prismatic aragonite crystals from (**m**).

Data on the hydrochemistry of Lake Van’s water is summarised elsewhere^14,19,20^ but is lacking a seasonal resolution. Contrasting interpretations of Lake Van carbonates have been summarised by McCormack et al.^14^. The most recent research identified inorganic low-Mg calcite and aragonite precipitating in the surface-water, biogenic low-Mg calcite (ostracod valves) and early diagenetic calcian dolomite (further referred to as dolomite, Fig. 1 and references therein). Here we document as yet unreported carbonate encrustations occurring solely around the valves of ostracods and other macroscopic biological remains. Encrusted ostracod valves are easily distinguished from well-preserved translucent valves and can be studied separately (Figs. 1, 2).

**Figure 3:**
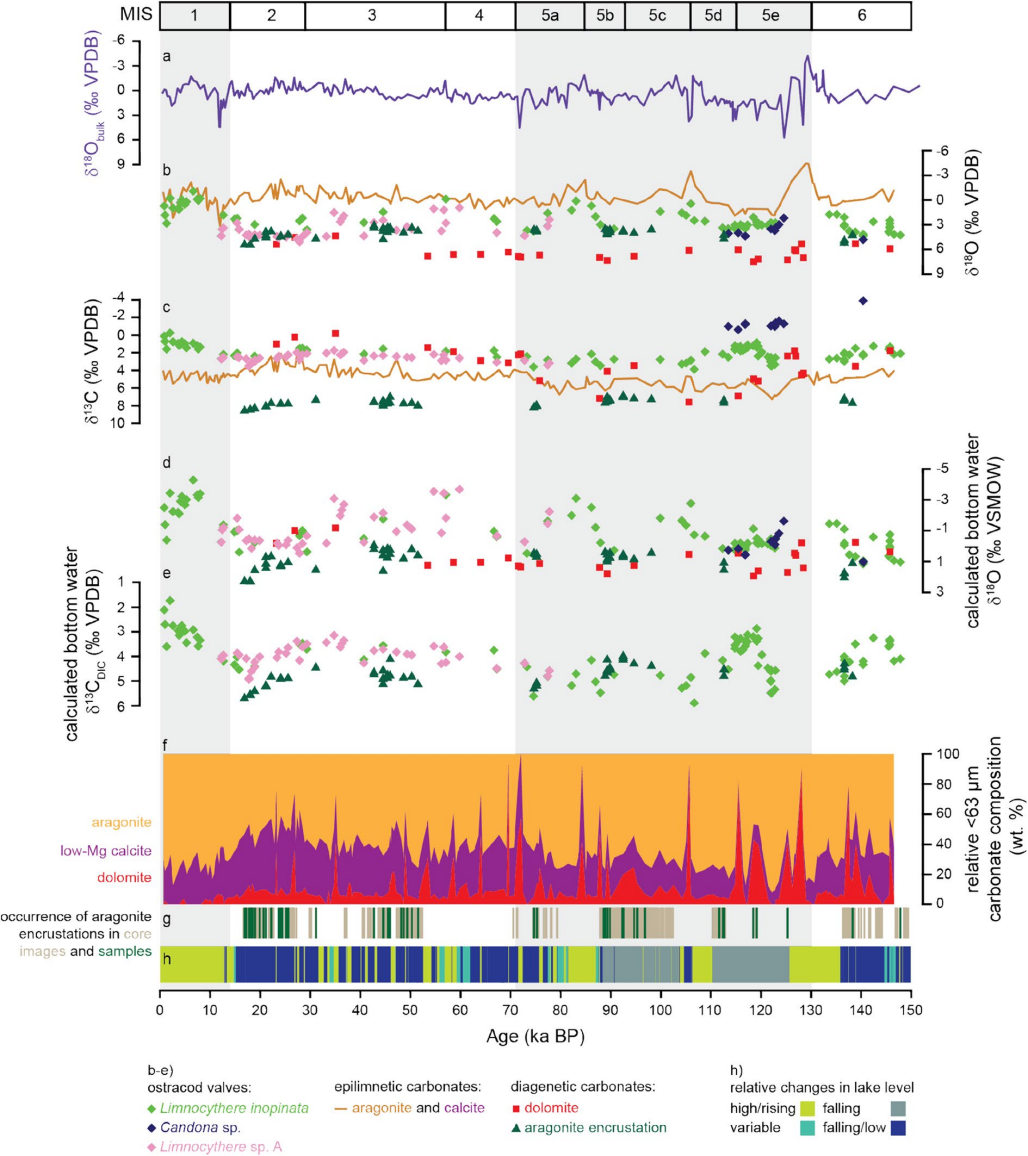
Isotopic variability in the Ahlat Ridge profile. (**a**) Bulk oxygen isotopy (δ^18^O_bulk_)^9^. (**b**) δ^18^O of different carbonate phases and (b) δ^13^C of different carbonate phases^14,25,31^. (**d**) Estimated bottom-water δ^18^O from deep-water carbonates, calculated for a constant temperature of 3.3 °C and using mineralogy specific fractionation factors (calcite^32^, aragonite^27^, dolomite^26^). For ostracod calcite a constant vital offset was assumed (0.8 ‰ for *L. inopinata*^35^, 2.2 ‰ for *Limnocythere* sp. A and 1.4 ‰ for *Candona* sp.^31^). (**e**) Estimated δ^13^C_DIC_ for epifaunal ostracod calcite and aragonite encrustations. Dolomite and *Candona* sp. are not depicted, as they likely incorporated carbon from pore-water DIC that varied isotopically from the bottom-water DIC. Estimations included a carbon isotopic offset from bicarbonate for calcite and aragonite of 1 and 2.7 ‰ respectively^48^ and a vital offset of 3 ‰ for *L. inopinata*^35^ and 2.4 ‰ for *Limnocythere* sp. A^31^. (**f**) relative carbonate composition^14^. (**g**) occurrence of aragonite encrustations from sieved samples (green) and anticipated from high-resolution core images (brown). (**h**) lithologies with genetic interpretations related to lake level variability as a coloured bar (simplified according to Stockhecke et al.^21^). Note that the isotopic variability between different carbonate phases from the same stratigraphic horizon is considerable, leading to an isotopically mixed signal in bulk measurements (**a**). Marine Isotope Stages (MIS) follow Lisiecki & Raymo^51^.

The encrustations are commonly visible to the naked eye as horizons of speckled dots in the sediment cores (Fig. 2a,b). This observation allows us to tentatively trace their occurrence, alone by carefully checking high resolution core images of the composite profile (Fig. 3g). As the images were taken directly after the cores were opened, we can exclude the formation of these encrustations at any point following that (i.e., during or after sample preparation). X-ray powder diffraction analyses and SEM observations (Fig. 2) confirmed that the composition of encrusted ostracod valves is dominated by aragonite with minor low-Mg calcite from the original valve. The aragonite crystals of the encrustation matrix are mostly anhedral or subhedral and intercalated with clay minerals if on the outside of ostracod valves or other remains (Fig. 2i,l). Larger subhedral to euhedral crystals formed within the space between valve and the encrustation (Fig. 2j,k). Even larger euhedral aragonite crystals formed within cavities, for example, of closed ostracod carapaces after decomposition of the soft tissue (Fig. 2m,n). Encrusted ostracod valves are often preserved with both valves articulated, however, in various stages of carapace opening, from fully closed to fully open (Fig. 2d–f). These encrustations occur in the sedimentary profile over broader intervals and are restricted to homogenous and banded muds (banded and mottled clayey silts), representing lake low-stands, reduced primary productivity/preservation and a well-ventilated water column^21^. There is no modern analogue in the lake for the banded lithotype^21^ nor any indication of encrustations occurring at the present. Within the studied interval aragonite encrustations appear intermittently in higher concentration but typically mutually exclusive from dolomite.

**Figure 4:**
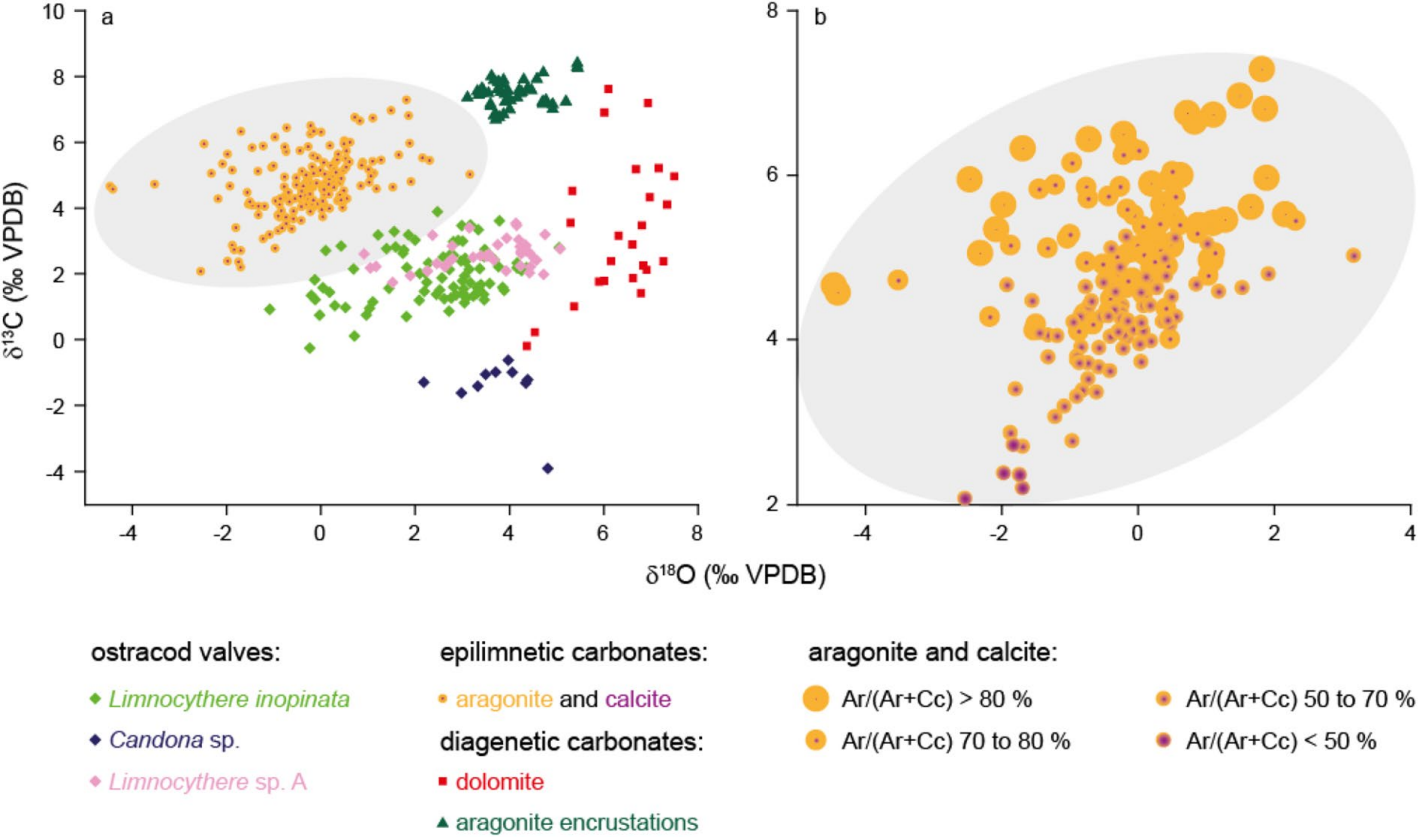
δ^13^C versus δ^18^O plot highlighting the isotopic variability between different carbonate phases in Lake Van. (**a**) Each carbonate phase plots in different fields suggesting different precipitation environments. Dolomite data from McCormack et al.^25^, epilimnetic aragonite and calcite data from McCormack et al.^14^ and ostracod isotopy from McCormack et al.^31^. (**b**) Isotopic variability between epilimnetic aragonite and calcite showing differences depending on the relative aragonite to calcite content (Ar/(Ar + Cc)) with grey area also depicted in (**a**).

Encrusted ostracod valves and hollow encrusted tubes (casts) from the same stratigraphic horizon are indistinguishable in their δ^18^O and δ^13^C values (Fig. 2g,h, Supplementary Table 1) suggesting that the isotopic signal contribution from the valve itself is negligible. Encrustations’ δ^18^O and δ^13^C values range between + 3.1 to + 5.4 ‰ and + 6.7 to + 8.4 ‰ respectively (Fig. 3). When including all Lake Van carbonate phases, the δ^18^O and δ^13^C values range between − 4.5 to + 7.5 ‰ for δ^18^O and − 3.9 to + 8.4 ‰ for δ^13^C (Fig. 3). Each carbonate phase has a distinct isotopic range, evidenced by their grouping in different fields on a δ^13^C versus δ^18^O plot (Figs. 1, 4). Fine fraction inorganic aragonite and low-Mg calcite cannot be easily separated and were analysed together (δ^18^O_Ar+Cc_, δ^13^C_Ar+Cc_) for dolomite-poor samples (< 15 volume % dolomite relative to calcite and aragonite in the fine fraction^14^).

## Discussion

Comparable (in appearance, quantity and episodic occurrence) aragonite encrustations were reported neither from Lake Van nor from any other lake sediments known to the authors. The aragonite encrustations occur exclusively on macroscopic biological remains (valves of ostracods or plant remains) or appear as hollow casts where their organic host was likely degraded with time (Fig. 2b to n). Their absence on lithoclasts (tephra, feldspar or quartz grains) leads us to postulate that their formation is linked to organomineralisation, i.e., is mediated by organic matter (OM), independently of the organism the OM derives from^22^. Consequently, the aragonite encrustations can be defined as a type of microbialite. Whether these microbialites are a product of active microbial metabolism (biologically-induced) or of passive mineralisation (biologically-influenced) remains, as of now, unresolved. Based on the preservation of articulated encrusted ostracod valves in various stages of carapace opening (Fig. 2d–f), i.e., documenting the taphonomic process of valve disarticulation, we assume a rapid initial encrustation process at work (hours to days), likely before burial in the sediment. In contrast, all non-encrusted ostracod remains are single disarticulated valves. Because the encrustation around and within biological remains takes place likely shortly after these have been “deposited” on the sediment surface, perhaps with some carbonate precipitation continuing during initial burial, we refer to them as an early diagenetic carbonate phase.

Alike all other carbonate phases in Lake Van’s sedimentary record, aragonite encrustations possess a distinctive isotopic signature (Fig. 4). The difference between the stratigraphic occurrence and the δ^18^O isotope range of inorganic fine fraction surface-water aragonite (calculated based on X-ray powder diffraction data)^14^ relative to aragonite encrustations strongly indicates a different origin of these two aragonite fractions (Fig. 4). Further, isotopic differences between all penecontemporary carbonate phases in Lake Van by far exceed any mineralogy-based water-carbonate fractionation factors alone (Figs. 3b,c, 4a). We postulate that these isotopic differences reflect true variations in the temperature (important for surface-water *versus* deep-water precipitates), oxygen isotope composition of the water (δ^18^O_water_) and carbon isotope composition of the dissolved inorganic carbon (δ^13^C_DIC_) of the individual precipitation environments. As such, the bulk isotope record of Lake Van represents a mixed signal^14^.

The strongest impact on the bulk carbonates isotopic composition comes from the volumetrically most abundant fine fraction; surface-water low-Mg calcite and aragonite or bottom-water diagenetic dolomite (Fig. 3a,b,f). Surface precipitates aragonite and calcite not only differ isotopically from the diagenetic dolomite but also from each other (Fig. 4b). Aragonite is enriched in ^18^O and ^13^C, beyond mineral-specific fractionation factors, relative to penecontemporary calcite^14^. Recognising the inherent technical challenges with fine fraction separation we focus on Lake Van’s bottom-water carbonate components which can be analysed individually; dolomite, ostracod valves and aragonite encrustations, and test their fidelity in recording environmental signals.

Although deep-water carbonate components do not always co-occur within the same stratigraphic layer, comparing and contrasting their respective isotopic compositions systematically throughout the profile allows a qualitative estimation of the palaeohydological conditions at the time of their formation. In case of deep-water carbonates precipitating below the thermocline, temperature changes are likely negligible. Based on lake level reconstructions^21,23,24^, the water column depth at the AR site was reaching at least 200 m during the last ca. 150 ka. If this is the case, variations in the isotopic composition of deep-water carbonate phases should primarily reflect changing bottom-water δ^18^O values and thus the evolution of Lake Van bottom-water over the last Glacial-Interglacial cycle.

### Oxygen isotopes in deep-water carbonates

The oxygen isotopic composition of aragonite encrustations (δ^18^O_encrustation_) varies between + 3.1 and + 5.5 ‰ (mean of + 4.09 ± 0.6 1SD). Similarly, dolomite δ^18^O values (δ^18^O_dolomite_) demonstrate a lower variability (+ 4.4 to + 7.5 ‰, mean of + 6.3 ± 0.8 1SD) compared to ostracod calcite and surface-water δ^18^O_Ar+Cc_ (Figs. 3b, 4a). In the Lake Van profile dolomite-rich intervals are discrete and commonly occur in finely laminated sediments representing lake high-stands, increased productivity/preservation and a an- or suboxic water column^25^; conditions apparently opposing those in which aragonite encrustations precipitate. Yet, dolomite formation is triggered after sediment deposition, within the sediment pore space^25^. A relatively narrow oxygen isotope range for both diagenetic phases implies that they precipitate only if certain hydrological conditions are met. The fact that both encrustations and dolomite are restricted to certain lithologies supports this hypothesis.

For the last 150 ka the calculation of the bottom-water oxygen isotope composition (δ^18^O_bw_) based on dolomite and encrustation δ^18^O (under the assumption of a constant temperature of 3.3 °C and applying mineral specific fraction factors^26,27^) gives a range of − 1.2 to + 2.3 ‰ VSMOW (Fig. 3d). This range covers the values measured for modern Lake Van water δ^18^O values (between − 0.4 and + 1‰ VSMOW)^28–30^. For the same interval, the alternative calculation of bottom-water values was based on ostracod δ^18^O (under the assumption of constant temperature of 3.3 °C) and applying constant vital offsets (0.8 ‰ for *L. inopinata*; 2.2 ‰ for *Limnocythere* sp. A and 1.4 ‰ for *Candona* sp.)^31^ before applying the calcite-water fractionation factor of Kim and O’Neil^32^. It appears that ostracod-based δ^18^O_bw_ values are in general more negative and have a wider range than those calculated from diagenetic carbonates (Fig. 3d). Noteworthy, diagenetic carbonates and non-encrusted ostracod calcite seldom co-occur in the same stratigraphic layer.

The comparison of our data highlights the current limitations of quantifying past water δ^18^O values in alkaline lakes. The inconsistencies in bottom-water values calculated from different carbonate fractions arise from a number of factors including limited understanding of the formation process of the diagenetic phases, possible kinetic effects such as precipitation rate influencing diagenetic carbonate-water fractionation factors, general uncertainties in carbonate-water fractionation factors^33,34^, and possibly larger than anticipated bottom-water temperature variability. Reconstructing δ^18^O_bw_ values based on ostracod δ^18^O values faces the challenge of still not fully understood species-specific vital effects perhaps depending on prevailing hydrochemical conditions^35–38^. The species analysed here differ in their δ^18^O and δ^13^C composition not only due to species-specific vital effects but also due to varying microhabitats^31^. Despite these interfering influences, the discreet and relatively low-resolution ostracod δ^18^O (and per extension the ostracod-based δ^18^O_bw_) record is the only carbonate oxygen isotope time series in Lake Van convincingly replicating hemispheric patterns of climate cyclicity^31^.

### Carbon isotopes in deep-water carbonates

Like the deep-water carbonate δ^18^O values, the δ^13^C values may indicate environmentally triggered changes in the lake water δ^13^C_DIC_ and/or provide information about the carbon source of the diagenetic phases, thus providing information regarding the timing, sedimentary depth and mechanisms of their formation. Potential vital offsets and/or moulting of species in different microhabitats^39^ can lead to significant differences between ostracod δ^13^C and the water column δ^13^C_DIC_. The epifaunal taxa in Lake Van display a relatively constant δ^13^C offset, with *Limnocythere* sp. A values approximately 0.6 ‰ higher than those of *L. inopinata*^31^. These species likely reflect bottom-water δ^13^C_DIC_ values modified by a species-specific vital offset. In contrast, infaunal *Candona* sp. has significantly lower δ^13^C values reflecting the δ^13^C_DIC_ of the pore water. Pore water δ^13^C_DIC_ is often shifted towards lower values due to the oxidation of ^13^C-depleted organic matter in the sediments. This pronounced carbon isotope difference between epifaunal and infaunal taxa implies a strong gradient between bottom-water and pore water δ^13^C values, when the oxic-anoxic boundary penetrates the uppermost sediments. From here, the wide range of dolomite δ^13^C values (from 0 to ca. + 8 ‰ VPDB, Figs. 3c, 4a) may be related to variable contributions of bottom and modified pore water δ^13^C_DIC_. The formation of dolomite in Lake Van was associated with physico-chemical perturbations of the pore water, connected to pronounced changes in the lake level^25^. Dolomite δ^13^C values may be linked to changes in penetration depth and/or intensity of these physico-chemical perturbations. This link may be direct, by supplying more or less lake water DIC into the sediment to mix with pore water DIC and/or indirect by leading to varying dolomite formation depths below the sediment–water interface or affecting microbial activities and/or their metabolic pathways.

As with ostracod calcite and early diagenetic dolomite, the δ^13^C values of aragonite encrustations (δ^13^C_encrustation_) can provide information about the timing and environment of the precipitation process. Encrustations occur exclusively in sediments related to a well-ventilated water-column during a lake-level lowstand. The generally low total organic carbon (TOC) content of these sediments reflects either low productivity or a high degradation of OM or both^21^. As encrusted ostracod valves appear often abundant within sedimentary layers (Fig. 2a,b), their presence supports well-oxygenated bottom-waters as well, because the high number of ostracod valves argues for a highly active benthos. The δ^13^C_encrustation_ values are significantly higher and more homogenous (+ 6.7 to + 8.4 ‰) compared to the δ^13^C values of *Candona* and dolomite (both formed in the pore water and not bottom-water and thereby likely utilised different DIC pool than the encrustations, Fig. 3c), thus precluding pore water δ^13^C_DIC_ as an important carbon source for encrustations. This hypothesis is also supported by the preservation of articulated encrusted ostracod valves in various stages of carapace opening (Fig. 2d–f) and by implication a rapid encrustation process.

Carbonates linked to the decomposition of organic matter and microbial respiration have typically lower δ^13^C values than expected from water column DIC equilibrium considerations. For example, decaying soft body tissues in marine carbonate concretions^40^, or the degradation of exopolymeric substances (EPS) by heterotrophic bacteria in freshwater microbialites^41^ can lead to a supply of isotopically light organic carbon at the carbonate precipitation site. The δ^13^C_encrustation_ values are ^13^C enriched relative to other carbonate phases in the lake sediments and thus do not unambiguously imply a biogenic origin of the carbon. However, biologically induced or influenced carbonate precipitation does not necessarily modify the carbonate carbon isotope composition, even if related to photosynthetic or respiratory microbial activity^42,43^. The inherently large DIC pool of Lake Van (~ 100 mmol kg^−1^)^20^ may dampen the impact of microbial activity on the DIC isotopic composition at the encrustation site.

**Figure 5:**
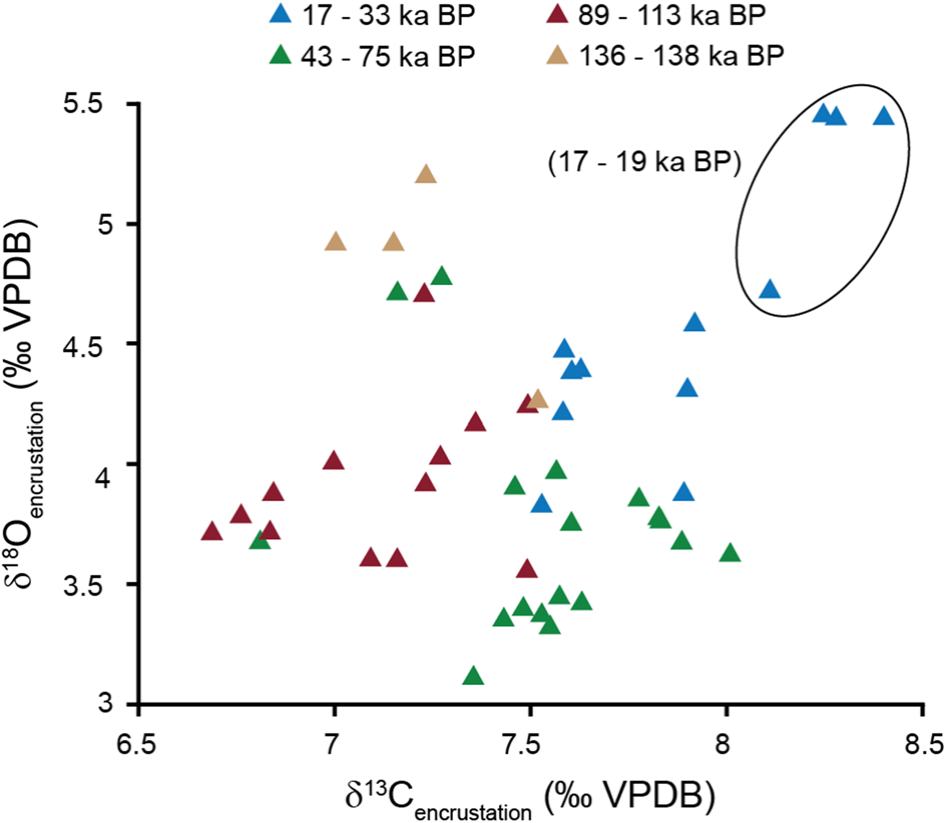
Isotopic variability within aragonite encrustations. Encircled samples are from the late Last Glacial Period (17 to 19 ka BP) exhibiting the highest δ^18^O_encrustation_ and δ^13^C_encrustation_ values. Minor differences can be observed in the oxygen and carbon isotopy of encrustations for different periods (different colouring), suggesting at least a partial environmental control on their isotopic signals.

Pedone and Folk^44^ suggested that the degradation of OM by bacteria in brine-shrimp eggs from Great Salt Lake catalysed aragonite cementation that was subsequently overgrown in a second cementation step by epitaxial, prismatic aragonite crystals. The growth and mineralisation of these prismatic aragonite crystals was assumed to continue inorganically on top of the biotic layer. In analogy, in a first step bacterial activity and the decay of OM may initiate the nucleation of aragonite on biological remains in Lake Van, followed by an inorganic growth of aragonite crystals. This scenario may explain the large prismatic aragonite crystals forming within the cavities of closed carapaces of the aragonite encrustations. Indeed, these encrustations represent single-crystal precipitates (Fig. 2k,l,n), which are typical for abiotically precipitating carbonates in low [Ca]/[Alkalinity] settings, observed in other soda lakes and experimentally^45^. This being the case, the isotopic composition of aragonite encrustations in Lake Van may mirror the bottom-water isotopy. Accordingly, the highest δ^13^C and δ^18^O values in aragonite encrustations are from the late Last Glacial period (19–17 ka BP, Fig. 5) where the lake level arguably reached its lowest level^21,24^. Aragonite encrustations from parts of MIS 5 (specifically 89 to 113 ka BP) also have generally lower carbon and oxygen isotope values than those from the late MIS 3 and MIS 2 (17 to 33 ka BP) perhaps related to more arid conditions during the later periods (Fig. 5). At this point, however, a contribution of carbon isotopically modified from water column DIC by microbial activity cannot be fully excluded.

If the δ^13^C_encrustation_ values indeed reflect bottom-water DIC values, one might expect similar values and/or trends in the δ^13^C record of ostracod calcite. δ^13^C_encrustation_ values are 4.6 to 6 ‰ higher compared to well preserved translucent valves of epifaunal ostracods from the same stratigraphic horizon (Fig. 3c). This significant enrichment in ^13^C in diagenetic aragonite relative to ostracod calcite cannot be explained by the differences in carbon isotope fractionation between aragonite and calcite, with aragonite being approximately 1.6 to 1.8 ‰ more positive than calcite when utilising the same DIC pool^46–48^. The impact of vital effects on the carbon isotope composition of ostracod calcite is even less understood than for oxygen. The carbon isotope values of *L. inopinata*, however, are suggested to be 1 to 3 ‰ lower than what is expected for equilibrium precipitation, a feature that may be attributed to this species specific biomineralisation pathway^35,39^. Combined, mineral-specific fractionation and vital effects may account for most of the variation between the δ^13^C of aragonite encrustations and ostracod calcite, an interpretation that at this point, however, is highly speculative. When adjusting for a carbon isotope offset from bicarbonate for calcite and aragonite of 1 and 2.7 ‰ respectively^48^ and a vital offset of 3 ‰ for *L. inopinata* (2.4 ‰ for *Limnocythere* sp. A)^31^, bottom-water DIC calculations from both ostracod valves and aragonite encrustations show similar values between 3 and 6 ‰ for most of the studied period (Fig. 3e). For MIS 5 and 6 δ^13^C_DIC_ estimated from ostracod calcite and aragonite encrustations are comparable (albeit rarely present in the same sample), but for MIS 2 and 3 mean encrusted aragonite δ^13^C_DIC_ shows an offset of approximately 1 ‰ from the mean DIC calculated from ostracod calcite (Fig. 3e). Such an offset may indicate changes in ostracod-DIC and/or encrusted aragonite-DIC carbon fractionation factors from MIS 5 and 6 to MIS 2 and 3, perhaps related to the significant increase in salinity and alkalinity during the later period^24^. In any case, both aragonite encrustations and ostracod calcite show an increase in bottom-water δ^13^C_DIC_ and higher δ^18^O values towards the Last Glacial Maximum (Fig. 3b,c) coherent with increased aridity, a lower lake level and a salinity maximum^21,24^.

## Conclusions

We present to date the most comprehensive suite of mineralogical and isotopic data measured on carbonates from sub-recent sediments of a still existing lake. Our data shed a new light on lacustrine early diagenesis. Different valve opening stages of encrusted ostracods suggest a suddenness of the encrustation process (hours to days). The presence of surface-water aragonite and low-Mg calcite in the fine fraction (documented by its distinct δ^18^O–δ^13^C cluster) of the same samples containing encrustations argue for a highly selective encrustation (i.e., aragonite precipitation) exclusively on biological remains. So far as we are aware, this is the first time that aragonite precipitated in the surface-water occurring stratigraphically coeval with early diagenetic aragonite have been convincingly documented from lacustrine sediments. Similarly, although encrustations and dolomite apparently occur in contrasting types of sediments, both diagenetic phases precipitate under oxic conditions (or when the oxygenation front reaches the sediment–water interface and topmost sediment). The lack of diagenetic carbonates in modern (Holocene) anoxic and finely laminated sediments of Lake Van supports this postulation. The juxtaposition of δ^18^O_bw_ calculated from different bottom-water carbonates from the same stratigraphic levels (under the assumption of the same, stable bottom-water temperature and considering mineral-specific fractionation factors) displays differences of up to 3 ‰ VSMOW within the same sample (Fig. 3d). Our results highlight the current limitations in quantifying past water δ^18^O values. These findings call for the determination of all carbonate components (water column and diagenetic) and their individual isotopic compositions before embarking upon environmental and palaeoclimate reconstructions.

## Methods

This study focusses on material recovered in 2010 in the frame of the International Continental Drilling Program PALEOVAN. We resampled 2-cm-thick intervals from the Ahlat Ridge composite profile and off-sections avoiding the sampling of event-deposits. Composite profile depth (and age)^49^ was assigned to off-section samples by visual correlation based on high resolution core images. The sampling range covers the uppermost 68 m (56 m without event deposits) of the composite profile corresponding to 147.8 ka BP with a mean temporal resolution of 54 years per sample. Sediment samples were wet-sieved into four fractions (> 250 µm, 250–125 µm, 125–63 µm, < 63 µm), rinsed with distilled water to avoid mineral precipitation after sieving and air dried. Details regarding the sample preparation, mineralogical and isotopic analyses of dolomite, surface-water (fine fraction) aragonite and calcite and well-preserved translucent ostracod valves are reported elsewhere^14,25, 31^.

Aragonite encrustations were picked under the stereomicroscope from the > 250 µm and 250–125 µm fractions and stored in microfossil slides. SEM images were taken from gold-sputtered samples using a LEO/Zeiss Gemini 1530 as well as a Gemini 2—Merlin, both operating with an acceleration voltage of 20 kV. The digital stereomicroscope photograph (Fig. 2b) was taken with a Zeiss Axiocam 105 color camera attached to a Stemi 508 stereomicroscope using the image software ZEN 2.3 lite. Aragonite was verified as the main carbonate phase of the encrustations based on crystal morphology under the SEM (Fig. 2) and X-ray powder diffraction analysis. X-ray powder diffraction analysis was performed, for two samples on isolated encrusted ostracod valves, using a PANalytical Empyrean equipped with a PIXcel1D detector, applying a tube voltage and current of 45 kV and 40 mA, respectively. The diffraction patterns were obtained from 5 to 65° 2θ using Cu Kα radiation with a step size of 0.0131° 2θ and a counting time of 1 s per step.

Carbon and oxygen isotope analyses of aragonite coatings were performed in continuous flow mode following the procedure described in Breitenbach and Bernasconi^50^ using a GasBench II coupled to a ThermoFinnigan MAT 253 mass spectrometer at the Ruhr-University Bochum. Up to 1000 μg (1 to 12 coated ostracod valves/hollow tubes) depending on the size of coated ostracod valves or tubes was weighed into borosilicate glass vials and oven-dried at 104 °C overnight. Samples were run at 70 °C for 1 h together with international standards NBS19, IAEA603, CO8. All results are reported with respect to the Vienna Pee Dee Belemnite (VPDB) standard unless otherwise indicated. The external standard deviation for oxygen and for carbon is < 0.07 ‰.

## Supplementary Information

Supplementary Information.

Supplementary Table.
