# Supplementary material for: Coeval primary and diagenetic carbonates in lacustrine sediments challenge palaeoclimate interpretations
Source: Sci Rep. 2021 Apr 12;11:7935. doi: 10.1038/s41598-021-86872-1 (PMC8041749; doi:10.1038/s41598-021-86872-1)
Supplement: Supplementary file 1 — Supplementary Information. [file 41598_2021_86872_MOESM1_ESM.pdf]

Supplementary Information for:

Coeval primary and diagenetic carbonates in lacustrine sediments challenge palaeoclimate interpretations

Authors: Jeremy McCormack & Ola Kwiecien

This PDF file includes:

Legend for Supplementary Table 1

Supplementary Table 1: Oxygen and carbon isotope composition of aragonite encrustations. Additional information includes depth in the Ahlat Ridge core, core section, depth in core section, age after Stockhecke et al.[49] and whether encrusted ostracod valves or encrusted hollow casts were measured (sheet 1 in the accompanying .xlsx file).
